# A Low-Noise Delta-Sigma Phase Modulator for Polar Transmitters

**DOI:** 10.1155/2014/521717

**Published:** 2014-02-25

**Authors:** Bo Zhou

**Affiliations:** School of Information and Electronics, Beijing Institute of Technology, Beijing 100081, China

## Abstract

A low-noise phase modulator, using finite-impulse-response (FIR) filtering embedded delta-sigma (ΔΣ) fractional-N phase-locked loop (PLL), is fabricated in 0.18 **μ**m CMOS for GSM/EDGE polar transmitters. A simplified digital compensation filter with inverse-FIR and -PLL features is proposed to trade off the transmitter noise and linearity. Experimental results show that the presented architecture performs RF phase modulation well with 20 mW power dissipation from 1.6 V supply and achieves the root-mean-square (rms) and peak phase errors of 4° and 8.5°, respectively. The measured and simulated phase noises of −104 dBc/Hz and −120 dBc/Hz at 400-kHz offset from 1.8-GHz carrier frequency are observed, respectively.

## 1. Introduction

Polar transmitters can achieve both high power efficiency and good linearity and become growing popular in modern wireless systems [1, 2]. Polar modulation utilizes envelope *A* and phase Φ components to represent the baseband symbols instead of the conventional *I*/*Q* format. A constant-envelope phase-only signal going through a phase-modulation path is multiplied with the signal envelope going through an envelope-modulation path in a switched-mode power amplifier (PA) to reconstruct the original baseband complex signal (*I* + *jQ*). The high power efficiency is achieved by using a nonlinear switched-mode PA to handle the constant-envelope phase-modulated RF signal, and the good linear transmission is accomplished by modulating the signal envelope through the supply voltage of the switched-mode PA [3].

**Figure 1:**
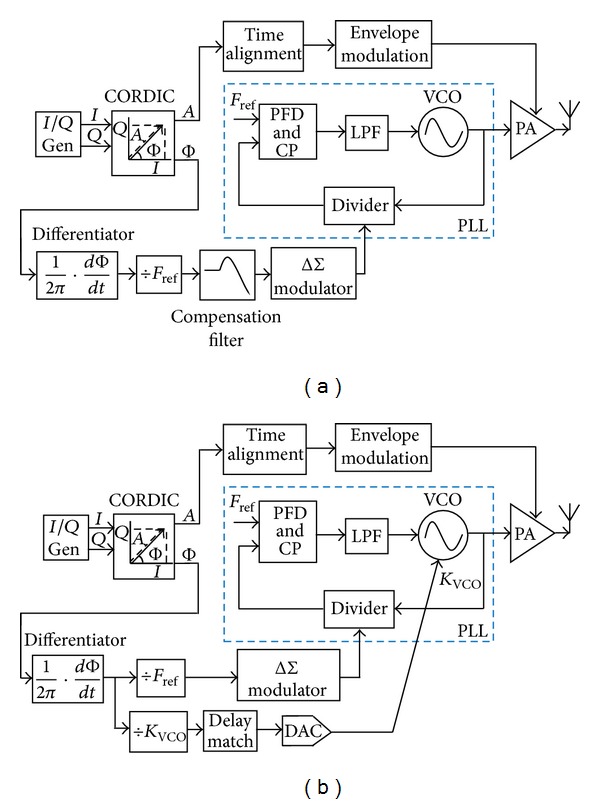
Polar transmitters with ΔΣ PLL based phase modulation: (a) digital precompensation and (b) two-point modulation techniques.

The delta-sigma (ΔΣ) fractional-N phase-locked loop (PLL) [4] enables phase modulation function when the fractional division ratio is modulated by the baseband signal, simplifying the overall transmitter architecture without requiring digital-to-analog converters (DACs) and RF upconverters [5].  Figure 1 shows the existing phase modulation methods based on fractional-N PLLs for polar transmitters. Since the conventional PLL bandwidth is not wide enough to accommodate the required modulation symbol rate, digital precompensation [6] or two-point modulation [7] techniques are employed to overcome the PLL bandwidth limitation but are prone to quantization noise jamming from the ΔΣ modulator (DSM).

The quantization noise qn of the DSM needs be concerned for a PLL. The existing noise cancellation technique [4, 8] requires good linearity and strict matching of the analog charge pump (CP) circuit, complicating circuit design. The reported finite-impulse-response (FIR) noise filtering method [9] effectively suppresses DSM noise but causes unexpected signal attenuation, when the DSM input is varying phase component rather than fixed.

In this paper, with simplified inverse-FIR and -PLL digital filters proposed to compensate for the signal attenuation, an FIR-embedded ΔΣ fractional-N PLL is presented to perform RF phase modulation for GSM/EDGE polar transmitters, achieving good trade-off between transmitter noise and linearity.

The paper is organized as follows. Section 2 presents the FIR-embedded ΔΣ phase modulation and clarifies the proposed FIR-compensated low-noise architecture. In Section 3, detailed design implementations are described and simplified inverse-FIR digital compensation filter is proposed, followed by experimental results in Section 4. Finally, conclusion is given in Section 5.

## 2. FIR-Compensated ΔΣ Phase Modulation for Polar Transmitters

### 2.1. FIR-Embedded ΔΣ Fractional-N PLL

**Figure 2:**
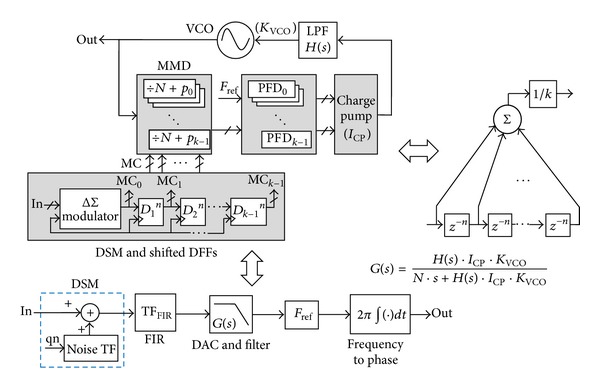
Conceptual diagram and signal model of FIR-embedded ΔΣ PLL with DSM noise suppression.

Figure 2 illustrates conceptual diagram and small-signal model of FIR-embedded ΔΣ fractional-N PLL. For the embedded FIR filter with a transfer function (TF) shown in (1), the numerator is performed by *k*-tap shifted D flip-flops (DFFs) with a delay depth of *n*, and by *k*-path multimodulus dividers (MMDs) and phase-frequency detectors (PFDs), and the denominator is done by a *k*-branch charge pump (CP) with *k*-phase inputs [9]. The multiple MMDs in parallel with sequential control bits from shifted DFFs perform FIR filtering on the DSM output

(1) $$\begin{array}{c} \text{T}{\text{F}}_{\text{FIR}}=\frac{\left(1+z^{-n}+z^{-2n}+z^{-3n}+\cdots +z^{-(k-1)n}\right)}{k}. \end{array}$$

Under PLL reference clock *F*_ref_, the notch frequency of FIR filter is located at multiples of *F*_ref_/(*n* × *k*)); thus the DSM output is conducted low-passed filtering. While the quantization noise qn is suppressed, the signal phase sent to the DSM is also attenuated with linearity degradation. Therefore, a digital compensation filter with inverse-FIR feature shown in (2) needs to be added before the DSM to offset the signal attenuation, without affecting the noise suppression. Obviously, high *n* × *k* value complicates the inverse-FIR filter design:

(2) $$\begin{array}{c} \text{T}{\text{F}}_{\text{Inverse-FIR}}=\frac{k}{\left(1+z^{-n}+z^{-2n}+z^{-3n}+\cdots +z^{-(k-1)n}\right)}. \end{array}$$

The signal path from the DSM input to the voltage-controlled oscillator (VCO) output, excluding the FIR filtering, is functionally equivalent to a DAC with a PLL TF *G*(*s*), followed by a multiplication factor *F*_ref_ and an integrator with frequency-phase conversion [10]. The PLL TF *G*(*s*) has low-passed feature with a limited signal bandwidth, which inversely degrades the phase-path bandwidth. Therefore, another digital compensation filter with inverse-*G*(*s*) feature shown in (3), a differentiator with phase-frequency conversion, and a divider-by-*F*_ref_ also should be added before the PLL to ensure the signal fidelity while phase modulation is performed:

(3) $$\begin{array}{c} \text{T}{\text{F}}_{\text{Inverse-PLL}}=\frac{1}{G\left(s\right)}=1+\frac{N\times s}{H\left(s\right)\times I_{\text{CP}}\times K_{\text{VCO}}}. \end{array}$$

### 2.2. Proposed Low-Noise Phase Modulator for Polar Transmitters

**Figure 3:**
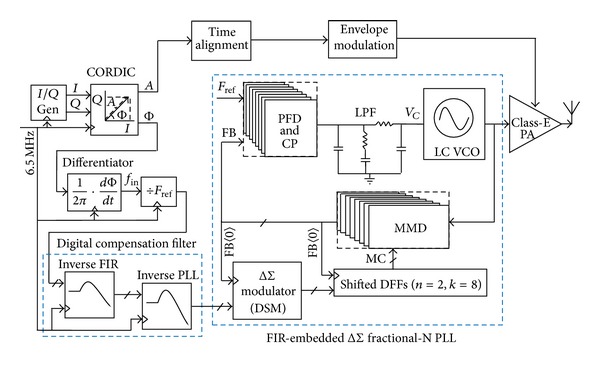
Proposed low-noise ΔΣ phase modulator with compensated-FIR filtering for polar transmitters.

Figure 3 gives the proposed low-noise delta-sigma phase modulator for polar transmitters, based on the discussion above. *I*/*Q* components of GSM/EDGE signals are generated based on a look-up table technique. The coordinate rotation digital computer (CORDIC) algorithm [11] accomplishes the conversion from *I*/*Q* to polar coordinate *A*/Φ with the original signal bandwidth of 200 kHz spread to more than 600 kHz. Here, the phase- and envelope-path bandwidths are set to 1 MHz.

Considering the PLL inherently contains a multiplication factor of *F*_ref_ and an integrator in function, a differentiator with phase-frequency conversion and a divider-by-*F*_ref_ are introduced. A digital filter having the equivalent inverse-*G*(*s*) TF shown in (3) compensates for the low-pass property of the PLL and widens the signal path. The other digital filter having the equivalent inverse-FIR TF depicted in (2) is employed to offset the gain attenuation by the FIR filtering.

A 4th-order type-II fractional-N PLL with FIR-embedded ΔΣ modulation and digital compensation filters is designed to perform RF phase modulation. Baseband digital phase is converted to RF analog phase by controlling the fractional division ratio via the DSM. In order to suppress the DSM noise, the FIR filtering with *n* = 2, *k* = 8 is chosen. With *F*_ref_ of 26 MHz, the notch frequency located at multiples of 1.625 MHz is achieved, which not only effectively suppresses the quantization noise but also does not complicate the inverse-FIR filter design.

## 3. Design Implementations

### 3.1. Baseband Component Generation

**Figure 4:**
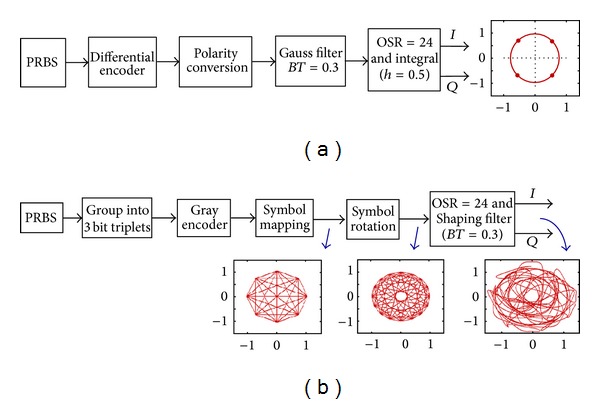
Block diagram of baseband *I*/*Q* signal generation: (a) GMSK for GSM and (b) 8PSK for EDGE.

Figure 4 gives the block diagram of GMSK based GSM and 8PSK based EDGE signal generation. Under symbol rate of 270.833 kSps, 6.5 MHz *I*/*Q* components are generated based on look-up table technique [12] where the tap coefficients of the 5-symbol-length Gaussian and shaping filters, with a BT value of 0.3, a modulation index *h* of 0.5, and an oversampling ratio (OSR) of 24, are stored in a read-only memory (ROM). A 2^21^−1 pseudo random bit source (PRBS) with a normal burst frame architecture produces the transmit data. The symbol rotation operation with 3*π*/8 phase shift ensures that the EDGE signal has the envelope component larger than 0.22 V, which lowers the nonlinear AM-AM and AM-PM distortions of the PA in polar transmitters.

The modified CORDIC [11] algorithm with low hardware cost is employed to accomplish the conversion from *I*/*Q* to polar coordinate *A*/Φ. The algorithm typically converges to a very small deviation after 10th or 11th iteration. Therefore, 16-times iteration is enough for the conversion resolution. For *i* = 1 and input vector (*I*, *Q*) from GSM/EDGE signal:

(4) $$\begin{array}{c} x_{2}=Q,\;\;y_{2}=-I,\;\;\theta_{2}=0.5. \end{array}$$

And the remaining iterations (for *i* = 2 ~ 16) are shown in (5), and finally, the desired phase and envelope components are given in (6):

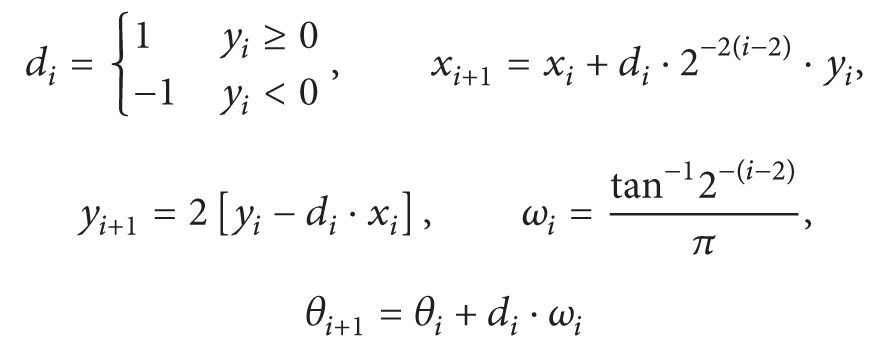
(5)

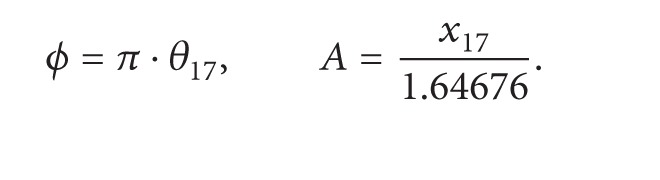
(6)


For the given five consecutive phase component inputs, a derivation algorithm using a five-point-interpolation method with phase jump preprocess is shown in (7) under a sampling clock of 6.5 MHz, to accomplish the phase-frequency conversion and generate the desired frequency component

(7) $$\begin{array}{c} f_{\text{in},k}=\frac{6.5\times 10^{6}}{24\pi }\left(\phi_{k-2}-8\cdot \phi_{k-1}+8\cdot \phi_{k+1}-\phi_{k+2}\right). \end{array}$$

**Figure 5:**
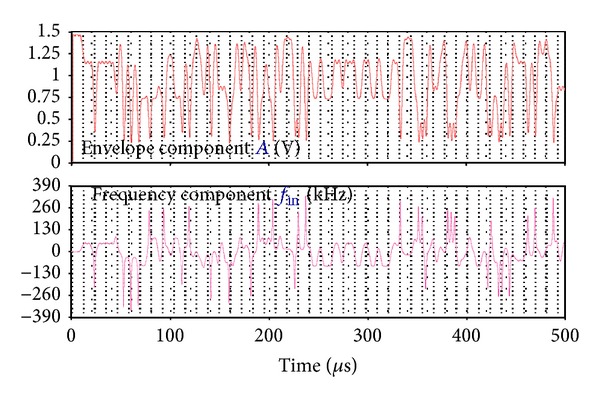
Simulated envelope and frequency components for EDGE signal.

Figure 5 gives the simulated baseband components for EDGE signal. Both the envelope component with an amplitude range of 0.22–1.44 V and the frequency component with a range of −340–340 kHz are observed. A peak frequency occurs when the envelope has a local minimum [2]. For GSM signal, both a constant envelope of 1V and a frequency range of −70–70 kHz are achieved. The frequency component divided by *F*_ref_ (26 MHz) is amplified by the sequent digital compensation filters with high-passed feature, which is still small enough for the DSM input not to cause the overflow.

### 3.2. Proposed Digital Compensation Filters

Since the fractional-N PLL has a low-passed feature with additional FIR attenuation, digital compensation filters need to be designed not only to compensate for the limited path bandwidth caused by the PLL but also to offset the gain attenuation due to the FIR filtering:

(8) $$\begin{array}{c} \text{T}{\text{F}}_{\text{Inverse-FIR}}=\frac{8}{\left(1+z^{-2}+z^{-4}+z^{-6}+z^{-8}+z^{-10}+z^{-12}+z^{-14}\right)}, \end{array}$$

(9) $$\begin{array}{c} \text{T}{\text{F}}_{\text{Inverse-FIR}}=\frac{2}{\left(1+\left(2.2/2.7\right)\cdot z^{-2}+\left(0.5/2.7\right)\cdot z^{-3}\right)}. \end{array}$$

**Figure 6:**
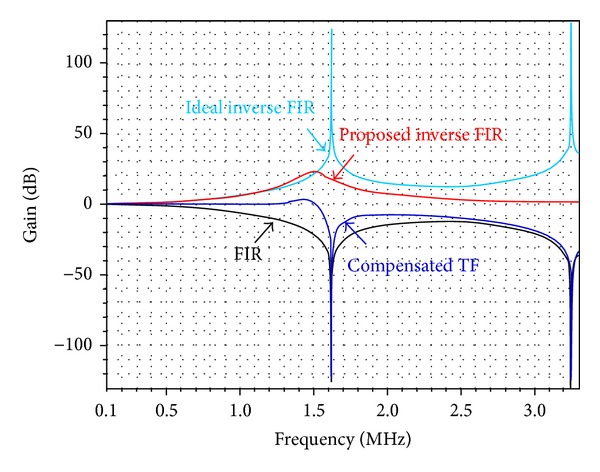
Simulated frequency responses of FIR-compensated filters: ideal versus proposed implementations.

Larger FIR parameter *n* or *k* contributes to lesser DSM noise but makes the inverse-FIR filter more complex. As a trade-off between the noise and complexity, *n* = 2, *k* = 8 is chosen. However, the ideal inverse-FIR filter conforming to (8), is prone to instabile response with 14th-order high-passed feature. Under 6.5 MHz sampling clock, a simplified 3rd-order filter is proposed in (9), which features unobvious high-passed or slight overshoot response but has the same property as the ideal inverse-FIR one within the desired frequency band up to 1 MHz. The circuit-level simulation results in frequency response for both ideal and proposed inverse-FIR filters are depicted in Figure 6. The proposed implementation is simple and stabile with slight overshoot but has the same compensation feature within desired frequency band, when compared to the ideal one.

**Figure 7:**
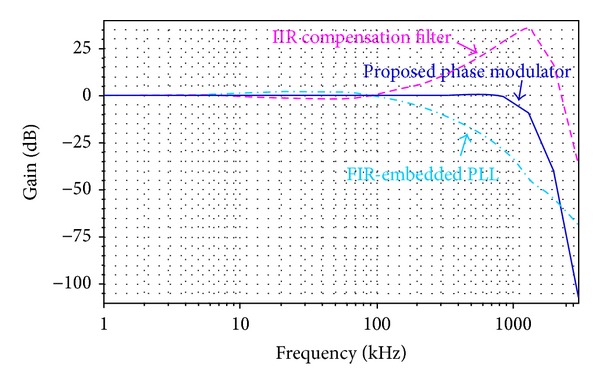
Simulated frequency response of presented phase modulator.

Since the type-II PLL with 4th-order TF inherently has 4 poles and 1 zero in *G*(*s*), accordingly, there are 4 zeros and only 1 pole existing in the inverse-*G*(*s*) TF. To ensure the compensation filter stable, additional 3 poles with farther locations are added to the inverse-*G*(*s*) filter, without affecting the frequency response within the desired frequency band. To further simplify filters, the proposed 3rd-order inverse-FIR and 4th-order inverse-*G*(*s*) filters are combined together by employing a 7th-order infinite-impulse-response (IIR) digital filter with a high-pass property.  Figure 7 shows the simulated frequency response of the presented phase modulator. For the PLL with a loop bandwidth of 100 kHz, the 7th-order IIR filter has a high-pass property with 100-kHz cut-off frequency and then rolls off at 1.3 MHz. As a result, the phase-path bandwidth of 1 MHz is achieved. The frequency component *f*_in_, divided by *F*_ref_ and then amplified by the IIR filter, is small enough for the DSM input not to cause overflow.

### 3.3. PLL Core Modules

To ensure phase-modulation linearity, it is important to ensure strict match of the TFs between the inverse-*G*(*s*) filter and the fractional-N PLL. Considering that the PLL TF is dependent on process, voltage, and temperature (PVT) variations, and the compensation filter is digitally fixed, thus, in most cases, the PLL loop-gain calibration or constant *I*_CP_ *K*_VCO_ techniques [13] need to be employed. However for the proposed design, the VCO gain *K*_VCO_ varies little under small control voltage range and the charge pump current *I*_CP_ is external reconfigurable, and the low-passed filter (LPF) of the PLL is off-chip and thus fixed. All these benefit the PLL TF robust over PVT.

**Figure 8:**
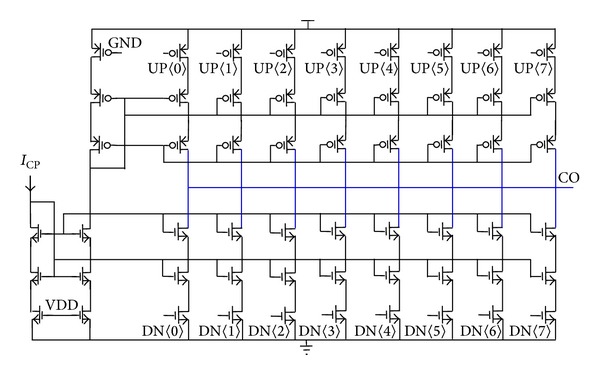
Multiphase charge pump schematic.

With embedded FIR filtering, the 4th-order type-II PLL with 100-kHz bandwidth employs eight PFDs in parallel, and all the lead-lag phase errors converted to push-pull pulse currents are summed at the output of the 8-phase charge pump. The schematic of the 8-phase charge pump is shown in Figure 8. Cascode biasing architecture helps to reduce switching noise from the multiple current switches and improves static and dynamic current match between the up and down branches. The current *I*_CP_ is external reconfigurable to ensure *I*_CP_ *K*_VCO_ constant.

**Figure 9:**
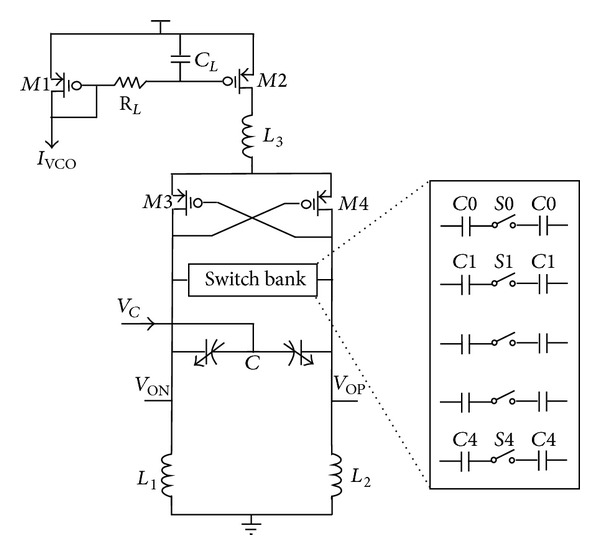
LC VCO schematic.

The summed push-pull error current is low-passed filtered to generate an error control voltage *V*_*C*_, which tunes the sequent VCO shown in Figure 9. The VCO employs the conventional cross-coupled three-transistor differential architecture with LC tank. The MIM switched capacitors having high quality factor are used for binary-weighted coarse tuning by a 5-bit control word to achieve ±15% frequency tuning range, while an NMOS accumulation-mode varactor is employed for fine frequency tuning by the input control voltage *V*_*C*_. An inductor *L*_3_ is inserted between the current source M2 and the cross-couple pair M3-M4 to reduce the second-order harmonic noise, and the embedded RC LPF suppresses the bias noise.

**Figure 10:**
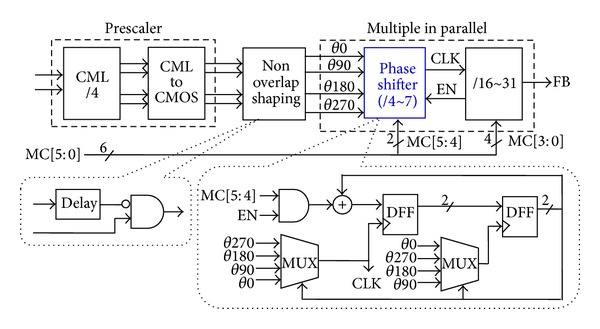
PS-based MMD module.

**Figure 11:**
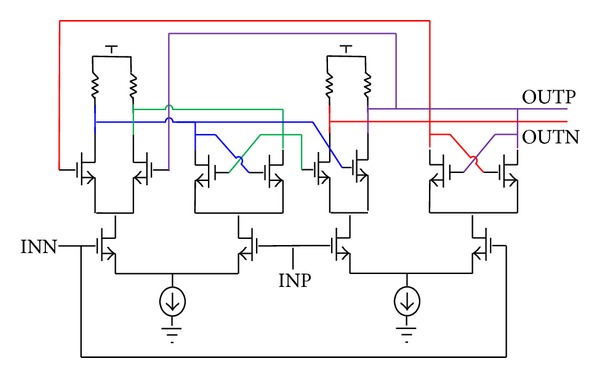
CML divider-by-2 schematic.

The VCO differential output is sent to the sequent MMD and compared to the reference clock.  Figure 10 shows phase shifter (PS) based MMD [9, 14] module to provide a programmable division ratio of 64–127 controlled by the baseband phase signal via the DSM. The high frequency current-mode-logic (CML) divider with a fixed division ratio of 4 generates quadrature output phases, which are shaped to nonoverlapping quadrature clocks to ease the timing requirement of logic circuits and are fed into eight parallel PS-based MMDs with 4-stage single-ended 2/3 prescalers supporting the division ratio of 16–31. The CML divider-by-4 is composed of two-stage cascaded dividers-by-2.  Figure 11 gives the schematic of the CML divider-by-2, which is similar to two-stage cross-coupled latch architecture.

Considering that fewer DSM output levels result in smaller instantaneous phase error at the PFD output and thus less phase noise, and on the other hand, the wider DSM output levels have more efficient randomization and dithering and generate less fractional spurs to the PLL [15], the DSM employs 4th-order single-loop architecture [14] as the trade-off between in-band noise and fractional spurs.

## 4. Experimental Results

**Figure 12:**
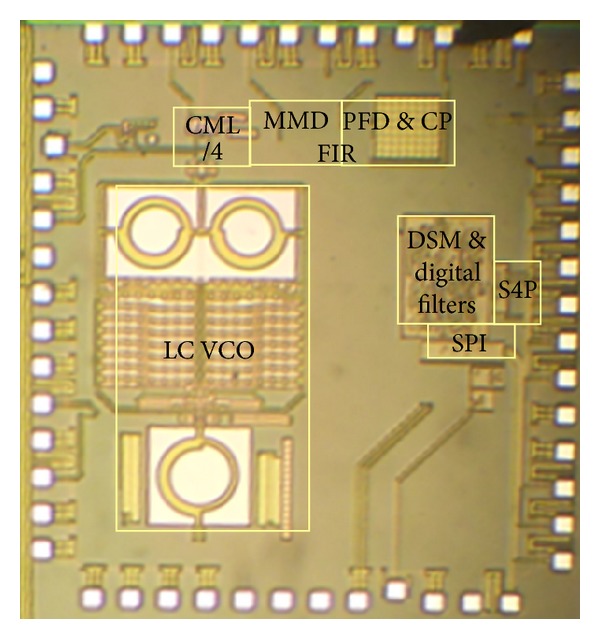
Chip micrograph.

The proposed digital compensation filters and FIR-embedded fractional-N PLL are fabricated in 0.18 *μ*m CMOS, and 16-bit baseband components are generated in a field programmable gate array (FPGA).  Figure 12 gives the chip micrograph with the active core area of 2 mm^2^ and the measured power dissipation of 20 mW from a 1.6 V supply, excluding the class-E PA. In order to save chip pads, on-chip 4-bit series-to-parallel conversion (S4P) and off-chip 4-bit parallel-to-series conversion (P4S) are used to accomplish the data communication between the chip and FPGA.

**Figure 13:**
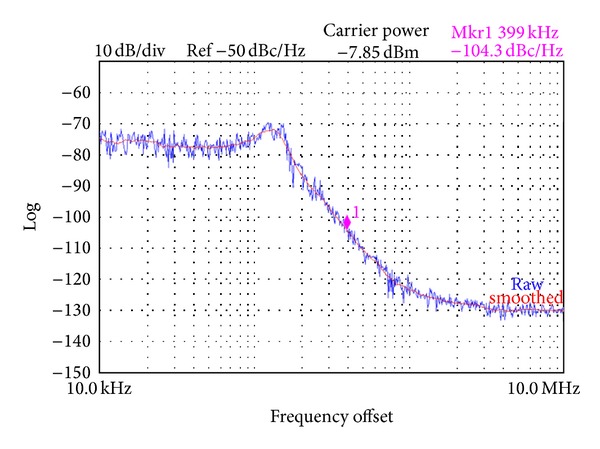
Measured phase noise of fractional-N PLL.

The simulated and measured phase noise depicted in Figure 13 of the fractional-N PLL, dominating the phase modulator system noise, is −120 and −104 dBc/Hz at 400-kHz offset frequency from the oscillating frequency of 1.8 GHz, respectively. The PLL bandwidth of 100 kHz is observed, which is compensated by the digital filters to ensure the phase-path bandwidth of 1 MHz. The measured tuning range and gain of the LC VCO centered at 1.8 GHz are ±15% and 5 MHz/V, respectively. The VCO has a small control voltage range of −70–70 mV, which contributes to small *K*_VCO_ variation with PVT.

**Figure 14:**
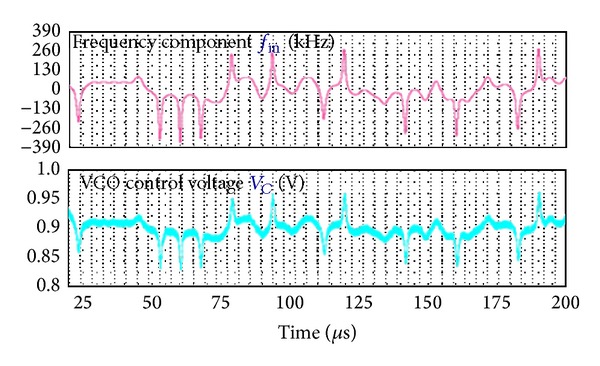
Simulated modulation performance of proposed architecture for 8PSK.

Figure 14 shows the simulated transient response of the proposed modulator for 8PSK signal. The phase modulation performance can be indirectly estimated by observing the tracking performance of VCO control voltage *V*_*C*_ with baseband frequency component *f*_in_. Both a fixed delay caused by the limited path bandwidth (1 MHz) and a fixed multiplication factor caused by the VCO gain *K*_VCO_ (5 MHz/V) are observed between the curves of *f*_in_ and *V*_*C*_ with similar waveforms. The presented architecture performs RF phase modulation well with slight phase ripples rising from little DSM quantization noise and VCO phase noise.

**Figure 15:**
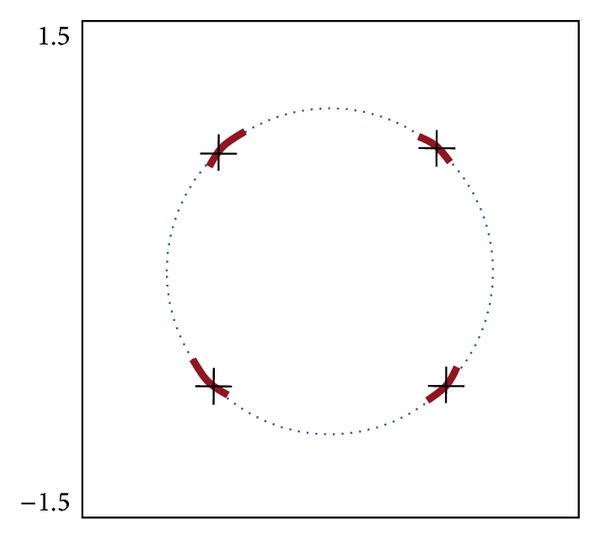
Transmitter output constellation for GMSK.

Figure 15 shows the transmitter output constellation for GMSK. The root-mean-square (rms) and peak phase errors are 4_rms_° and 8.5_peak_°, respectively, meeting error vector magnitude (EVM) requirements [16] of GMSK. Due to little nonlinearity caused by slight TF mismatch between the compensation filter and PLL, the deviation of the symbol vectors from the four homocentric points is observed in the constellation. The loop gain autocalibration technique [13], as discussed above, ensuring *I*_CP_ *K*_VCO_ constant and thus TF strict matching, may be considered to further optimize the modulation linearity.

## 5. Conclusions

A phase modulator employing FIR-compensated ΔΣ PLL is fabricated in 0.18 *μ*m CMOS with baseband components generated by FPGA. Digital compensation filters are proposed to trade off the noise and signal integrality. The experimental results show that the presented architecture performs RF phase modulation correctly with good noise and linearity performances.
